# Data of dynamic microscale strain distributions of Ti-6Al-4V alloys in dwell fatigue tests

**DOI:** 10.1016/j.dib.2019.104338

**Published:** 2019-08-13

**Authors:** Qinghua Wang, Shien Ri, Akira Maenosono, Yoshihisa Tanaka, Motomichi Koyama

**Affiliations:** a National Metrology Institute of Japan, National Institute of Advanced Industrial Science and Technology, 1-1-1 Umezono, Tsukuba, Ibaraki 305-8568, Japan; b Department of Mechanical Engineering, Kyushu University, 744 Motooka, Nishi-ku, Fukuoka, Fukuoka, 819-0395, Japan; c Research Center for Structural Materials, National Institute for Materials Science, 1-2-1 Sengen, Tsukuba, Ibaraki 305-0047, Japan

**Keywords:** Fatigue, Creep, Strain, Plastic deformation, Scanning electron microscope, Image processing

## Abstract

Dynamic microscale strain distributions with temporal resolution of 1 s in a smooth and a cracked Ti-6Al-4V alloys during one-cycle dwell fatigue tests are illustrated in videos (URL: https://drive.google.com/drive/folders/1pit_VV2apGOpETVfaJAAtL5Xl2CNOiJ3?usp=sharing). The tensile strain distributions were measured by the video sampling moiré method from the 1-μm-pitch grid images in a scanning electron microscope. The strain concentration factors of the smooth and the cracked specimens are 1.96 and 2.65, respectively. The plastic strain increment is 0.0007 during the displacement holding time of 591s in the smoothed specimen at maximum stress of 900 MPa., and 0.0008 during the displacement holding time of 593s in the cracked specimen at maximum stress of 870 MPa. The typical strain results are analyzed in 1-s-resolved strain mapping in Ti-6Al-4V alloys during dwell fatigue in SEM by video sampling moiré [1].

**Table:** Specifications Table

Subject area	*Physics*
More specific subject area	*Optics, Materials physics, Engineering physics*
Type of data	*Figure, Videos*
How data was acquired	*Scanning Electron Microscope (SEM), Image processing*
Data format	*Raw, analyzed*
Experimental factors	*Specimens were mechanically ground and polished.*
Experimental features	*Dynamic SEM image recording during one-cycle dwell fatigue tests*
Data source location	*National Metrology Institute of Japan, National Institute of Advanced Industrial Science and Technology, Tsukuba, Ibaraki, Japan*
Data accessibility	In a Google Drive public repository and this article URL: https://drive.google.com/drive/folders/1pit_VV2apGOpETVfaJAAtL5Xl2CNOiJ3?usp=sharing Folder name: Dynamic strain distributions of Ti-6Al-4V alloys Data identification number: Video 1, Video 2, Image folder 1
Related research article	Q. Wang, S. Ri, A. Maenosono, Y. Tanaka, M. Koyama, 1-s-resolved strain mapping in Ti-6Al-4V alloys during dwell fatigue in SEM by video sampling moiré, Mech. Mater. 133 (2019) 63–70.

**Table:** 

**Value of the data** • The experimental strain distributions can serve as a benchmark for numerologists to establish reasonable simulation models. • The dynamic strain maps can be combined with electron backscatter diffraction results for clarifying the failure mechanisms of Ti alloys. • These plastic strain increments and the relevant measurement method can provide references for strengthening and toughening of various materials. • The strain videos and the video sampling moiré method offer a choice of method for dynamically visualizing strain and stress around cracks for materials and mechanics experts.

## 1 Data

**Fig. 1:**
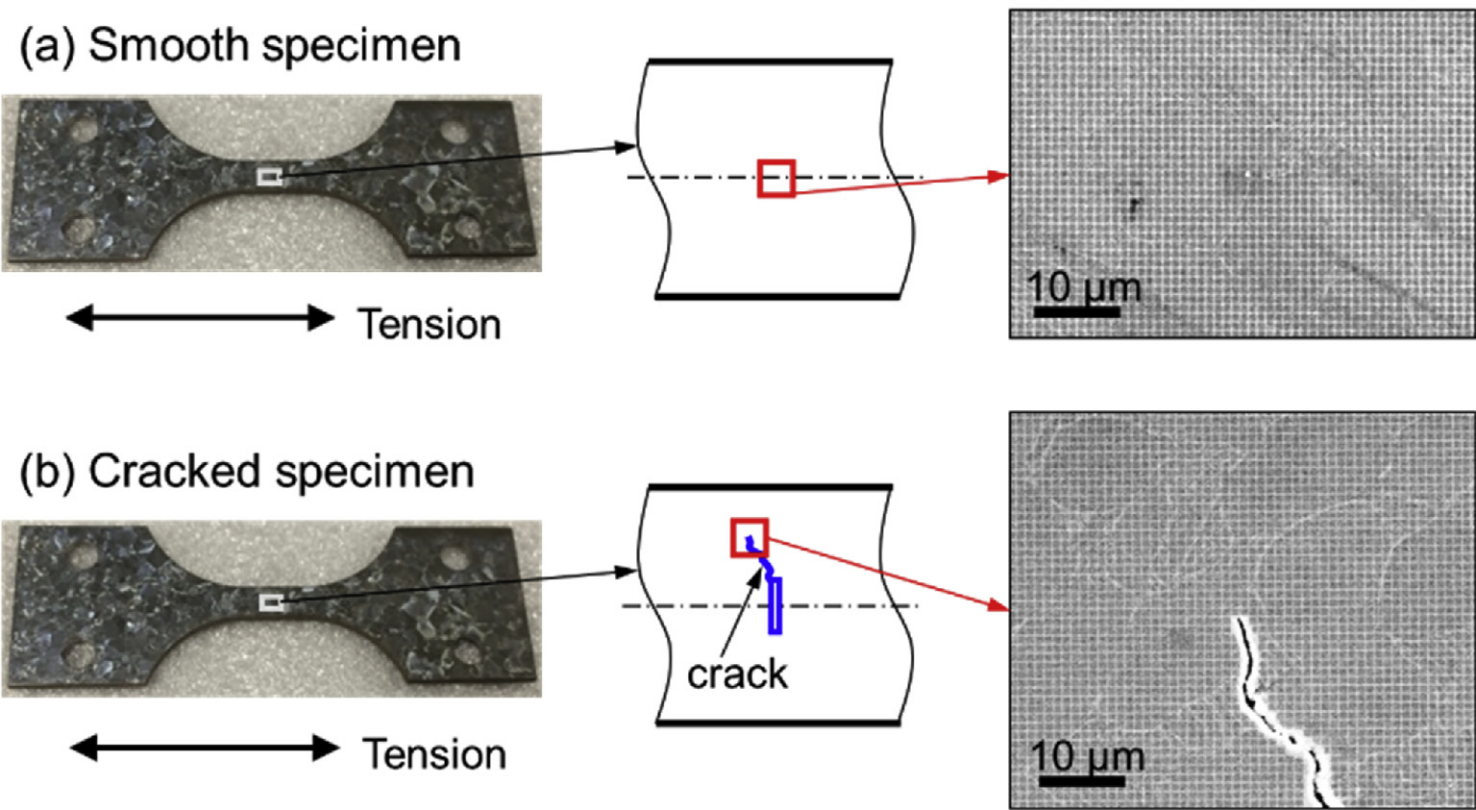
Locations of analyzed areas in Ti-6Al-4V alloy specimens and 1-μm-pitch grid images.

**Fig. 2:**
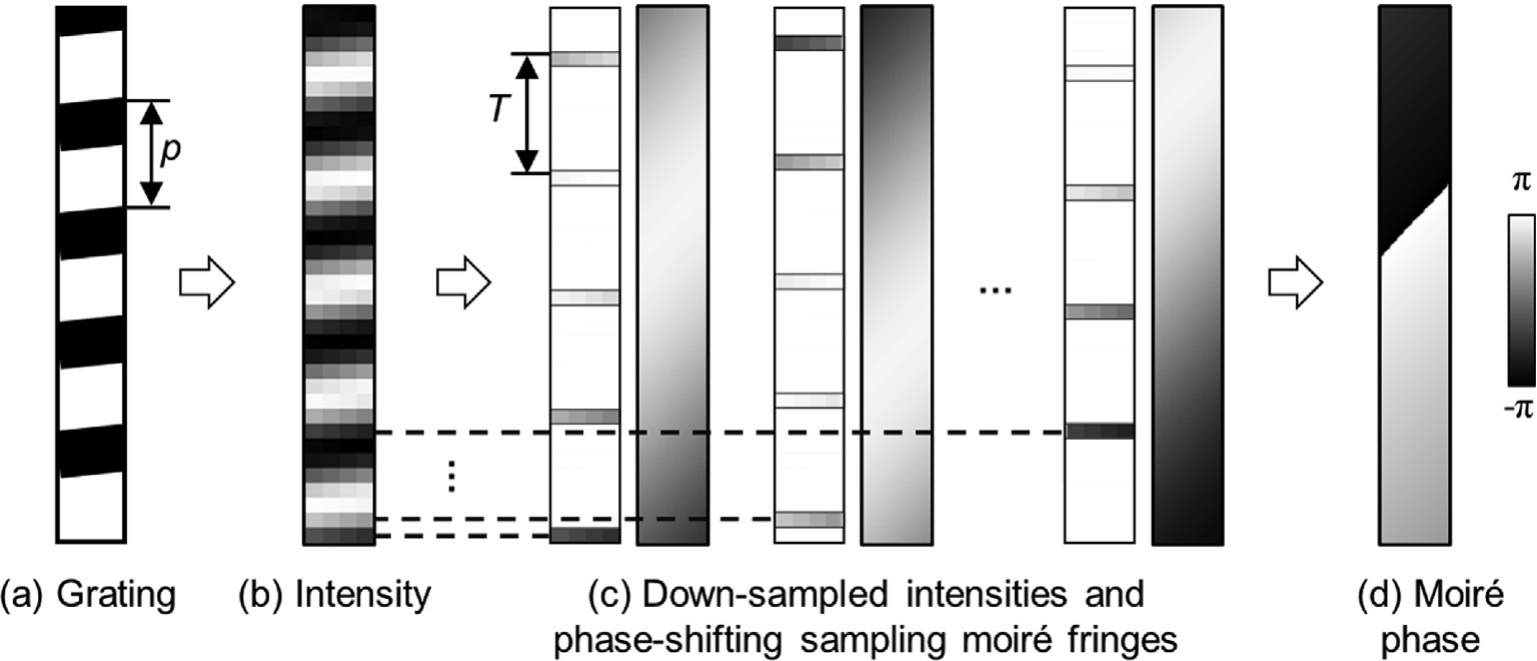
Moiré generation process and phase measurement principle of the sampling moiré method, where *p* is the grating pitch and *T* means the sampling pitch.

The shared data are 1-μm-pitch grid images and the 1-s-resoved strain results on Ti-6Al-4V alloys during dwell fatigue tests (URL: https://drive.google.com/drive/folders/1pit_VV2apGOpETVfaJAAtL5Xl2CNOiJ3?usp=sharing). The analyzed grid images were located at the center of a smooth Ti-6Al-4V alloy, and near a crack tip originated from a notch artificially made at the center of a cracked Ti-6Al-4V alloy (Fig. 1), respectively. The strain distribution in the tensile direction of either specimen was calculated from phase analysis of the spatial phase shifting sampling moiré fringes generated from a single grid image (Fig. 2) at each moment. Video 1 shows the dynamic strain distributions in a smooth Ti-6Al-4V alloy in one-cycle dwell fatigue test with maximum tensile stress of 900 MPa. Video 2 presents the dynamic strain distributions in a cracked Ti-6Al-4V alloy in one-cycle dwell fatigue test with maximum tensile stress of 870 MPa. In Videos 1 and 2, X indicates the tensile direction and the scale is 10 pixels/μm. Image folder 1 depicts the original strain distributions of the cracked Ti-6Al-4V alloy, where the strain concentration occurs above the crack tip at the maximum load and almost disappears after unloading. Table 1 lists the loading parameters, and the summarized strain results in 54 × 38 μm^2^ of the smooth specimen and 54 × 20 μm^2^ above the crack tip of the cracked specimen.

**Table 1:** Loading parameters and strain results of Ti-6Al-4V alloys in dwell fatigue tests.

Specimen	Smooth	Cracked
Loading Speed (MPa· s^−1^)	18	11
Maximum tensile load (MPa)	900	870
Displacement holding time (s)	591	593
Fluctuation of average strain during dwell	0.0082–0.0096	0.0140–0.0154
Fluctuation of maximum strain during dwell	0.0148–0.0251	0.0290–0.0498
Fluctuation of maximum strain with 10s filter during dwell	0.0173–0.0189	0.0334–0.0438
Fluctuation of strain concentration factor	1.90–2.11	2.33–2.94
Mean of strain concentration factor	1.96	2.65
Growth rate of average strain (s^−1^)	1.1 × 10^−6^	1.4 × 10^−6^
Growth rate of maximum strain (s^−1^)	2.6 × 10^−6^	1.4 × 10^−5^
Average strain increment during dwell	0.0007	0.0008
Unloading speed (MPa· s^−1^)	16	13
Plastic strain after 1-cycle test	0.0010	0.0014

The following is/are the supplementary data related to this article:

Video 1 1 Video 1

Video 2 2 Video 2

## 2 Experimental sdesign, materials and methods

### 2.1 Materials and grid fabrication

The specimen material was Ti-6Al-4V bimodal titanium alloy [2]. A smooth specimen and a cracked specimen were prepared by mechanical polishing. 1-μm-pitch grids were fassbricated on both specimens by electron beam lithography. The electron beam resist (EBR-9, Toray) was spin-coated on either specimen at 2000 rpm for 60s, baked at 195 °C for 30 min [3], and then exposed with electron beam in a scanning electron microscope (SEM, Quanta 200 FEG) combined with a patten generator (SPG-724, Sanyu Electron). Then, the resist was developed in a Type 1 solution for 60 s and rinsed in a 2-propanol solution for 30 s to generate a grid pattern on either specimen. To ensure SEM observation without the trouble caused by electrostatic discharge, the resist grid pattern was covered by a very thin gold layer using an ion coater (IB-5, EIKO Engineering).

### 2.2 Dwell fatigue test

The specimen size, the one-cycle 10-min dwell fatigue tests, and the dynamic grid image recording were detailly described in Ref. [1]. The dwell fatigue experiments were performed in anther SEM (JEOL IT300) equipped with an in-situ tensile stage (Gatan Mtest5000). The maximum tensile stress was 900 MPa and 870 MPa for the smooth and cracked specimens, respectively. The scanning speed was 1 image per second at a resolution of 640 × 480 pixels.

### 2.3 Strain measurement

The strain analysis areas on both specimens were located at the specimen centers as seen in Fig. 1. In the dynamic grid images of either specimen, the grid pitch was around 10 pixels. The dynamic strain distributions in the tensile direction were calculated from the grid images by the developed video sampling moiré method. The recorded video images (10 fps) were sampled over time according to the SEM scanning speed (1 fps), and then the selected images by time sampling were analyzed by spatial sampling moiré.

To reduce the influence of heavy noise on the strain measurement, multi-stage filtering was used in multiple calculation steps and the detailed filter sizes were introduced in Ref. [1]. Each grid image was first used to extract vertical grating perpendicular to the tensile direction by a low pass filter with half the length of 10 pixels. From each extracted grating, the sampling pitch was set as 10 pixels and 10-step sampling moiré fringes were generated. The moiré generation process [4], [5] from a grating and the corresponding moiré phase is presented in Fig. 2. Each moiré phase was calculated by a spatial phase shifting technique. Then each strain distribution was calculated from the differential of the phase difference before and after deformation using a local phase unwrapping algorithm [6].

### 2.4 Video production and numerical analysis

The measured dynamic strain distributions, and the corresponding SEM grid images as well as the loading curves in the smooth and cracked specimens at the time scale of 1 s, were made into two videos at the frame rate of 10 fps in Matlab, as shown in Videos 1 and 2. The typical strain distributions and the analysis were shown in Ref. [1].

The strain concentration factor in Table 1 were calculated from the maximum strain divided by the average strain, where the maximum strain was smoothed by a time filter of 10s to attenuate the effect of heavy noise on the maximum strain fluctuation. The growth rates of the average and maximum strains in Table 1 were obtained by linear fitting of the average strain and the smoothed maximum strain, respectively.

## References

[1] Wang Q., Ri S., Maenosono A., Tanaka Y., Koyama M. (2019). 1-second-resolved strain mapping in Ti-6Al-4V alloys during dwell fatigue in SEM by video sampling moiré. Mech. Mater..

[2] Koyama M., Tanaka Y., Tsuzaki K. (2018). Micrographic digital image correlation coupled with microlithography: case study of strain localization and subsequent cracking at an FIB notch tip in a laminated Ti-6Al-4V alloy. Exp. Mech..

[3] Kishimoto S., Xing Y., Tanaka Y., Kagawa Y. (2008). Measurement of strain and stress distributions in structural materials by elcetron moire method. J. Solid Mech. Mater. Eng..

[4] Wang Q., Ri S., Tsuda H., Koyama M., Tsuzaki K. (2017). Two-dimensional Moiré phase analysis for accurate strain distribution measurement and application in crack prediction. Opt. Express.

[5] Ri S., Fujigaki M., Morimoto Y. (2010). Sampling moiré method for accurate small deformation distribution measurement. Exp. Mech..

[6] Wang Q., Ri S., Tsuda H., Koyama M. (2018). Optical full-field strain measurement method from wrapped sampling Moiré phase to minimize the influence of defects and its applications. Opt. Lasers Eng..

